# Antibacterial Mechanism of Allicin E Against *Aeromonas hydrophila* and Therapeutic Effect in *Carassius auratus gibelio*

**DOI:** 10.3390/antibiotics15040377

**Published:** 2026-04-08

**Authors:** Jinlong Li, Liushen Lu, Kai Chen, Ting Qin, Jun Xie, Ping Fang, Bingwen Xi

**Affiliations:** 1 Wuxi Fisheries College, Nanjing Agricultural University, Wuxi 214081, China; 2023813055@stu.njau.edu.cn (J.L.); luliushen@ffrc.cn (L.L.); chenk@ffrc.cn (K.C.); qint@ffrc.cn (T.Q.); xiej@ffrc.cn (J.X.); 2 Key Laboratory of Aquatic Animal Nutrition and Health, Freshwater Fisheries Research Center, Chinese Academy of Fishery Science, Wuxi 214081, China; 3 Freshwater Fisheries Research Institute of Jiangsu Province, Nanjing 210017, China; fang_ping@126.com

**Keywords:** allicin E, *Aeromonas hydrophila*, traditional medicine, antibacterial activity, *Carassius auratus gibelio*

## Abstract

**Background/Objectives**: The frequent use of antibiotics has led to increasing drug resistance in *Aeromonas hydrophila*; therefore, there is an urgent need to develop novel antimicrobial agents to prevent and control bacterial diseases in aquaculture. Allicin E (ALE) is derived from garlic (*Allium sativum* L.), a plant extensively used in traditional medicine for treating infections. This study aimed to evaluate the potential of ALE against *A. hydrophila*, a major aquaculture pathogen, by investigating its antibacterial efficacy, mechanisms of action, and in vivo protective effects. **Methods**: The minimum inhibitory and bactericidal concentrations (MIC/MBC) were determined by broth microdilution. Antibacterial mechanisms were investigated through ROS detection, electron microscopy, fluorescent staining, and content leakage measurement. In vivo efficacy was evaluated in *Carassius auratus gibelio* by monitoring survival rates and bacterial loads, analyzing immune and antioxidant biomarkers, and histopathological analysis after *A. hydrophila* challenge. **Results**: ALE exhibited potent antibacterial activity (MIC = MBC = 8 μg/mL), achieving complete bacterial elimination within 1 h and showing a low resistance propensity. Mechanistically, ALE induced ROS accumulation, causing oxidative damage that disrupted membrane integrity and facilitated the leakage of cellular contents. In vivo, ALE significantly enhanced fish survival, reduced bacterial loads, modulated inflammatory cytokines, boosted antioxidant enzyme activities (SOD and CAT), and alleviated tissue damage. **Conclusions**: ALE possesses potent in vitro antibacterial activity and exerts an inhibitory effect on bacteria-induced inflammatory responses, effectively combating *A. hydrophila* through a multi-target mechanism and enhancing host resistance.

## 1. Introduction

Motile Aeromonas Septicemia, primarily caused by *Aeromonas hydrophila*, is a pervasive disease in global freshwater aquaculture [1]. Gibel carp (*Carassius auratus gibelio*) is one of the most economically important freshwater aquaculture fish species in China, and bacterial septicemia caused by *A. hydrophila* is one of the most harmful diseases in gibel carp farming [2]. It inflicts severe economic losses on annual freshwater farming, characterized by high morbidity and mortality across a wide range of susceptible species, including fish, amphibians, and reptiles [3,4]. *A. hydrophila* is a typical opportunistic pathogen ubiquitous in freshwater environments [5,6,7]. It can exist as a commensal organism in the gastrointestinal tracts and on gills of healthy fish. However, aquaculture stressors, such as poor water quality and high stocking density, can trigger the outbreaks of disease caused by *A. hydrophila* [8]. While antibiotics including enrofloxacin and florfenicol are commonly employed to control such infections, their widespread and frequent use has led to the emergence of antibiotic-resistant Aeromonas strains, which has brought a serious problem to the clinical treatment and fish product safety [9,10]. This trend poses a serious threat to disease management by jeopardizing both clinical treatment and the effectiveness of aquaculture pharmaceuticals. Therefore, there is an urgent need to develop novel antimicrobial agents to prevent and control bacterial diseases in aquaculture.

**Figure 1:**
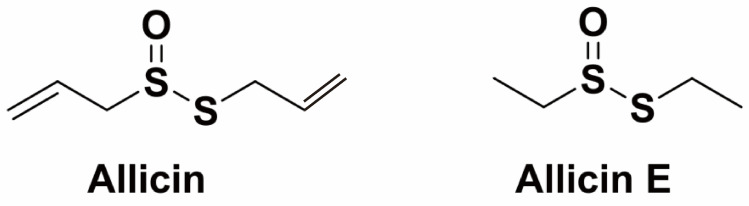
Chemical structures of allicin and ALE.

Addressing this challenge, bioactive compounds from Chinese herbal medicine (CHM) offer a promising alternative for managing bacterial diseases [11,12]. CHMs have been used for millennia to treat various diseases in both human and animal health, renowned for their broad-spectrum antimicrobial activity, lower incidence of resistance, and eco-friendly properties. Unlike many antibiotics that act on a single target, CHM-derived bioactive compounds can interfere with multiple bacterial pathways concurrently [13]. This multi-target mechanism is crucial for overcoming resistance and effectively countering complex virulence strategies, such as biofilm formation [14,15]. Consequently, the exploration of CHM applications in aquaculture has gained significant momentum as a strategic approach to reducing antibiotic dependence. Among various phytogenic candidates being investigated, garlic (*Allium sativum* L.), a key component in many traditional Chinese medicinal formulas historically used for treating infections and inflammation, is a prime example. Its bioactive compounds, allicin and its derivatives like allicin E (ALE), are responsible for many of its therapeutic properties [16,17]. The chemical structures of allicin and ALE are shown in Figure 1. ALE, an organosulfur compound derived from the bioactive compound (allicin) formed from crushed garlic, is widely recognized for its potent antibacterial properties [18,19]. Notably, evidence indicates that ALE employs a distinct multi-faceted antibacterial strategy, specifically involving membrane disruption and oxidative stress induction [20]. However, its efficacy and precise mechanism of action against key aquaculture pathogens such as *A. hydrophila* remain inadequately elucidated.

This study aims to comprehensively evaluate the potential of ALE as a novel therapeutic agent against *A. hydrophila*. We systematically investigate its direct antibacterial efficacy, inhibition of biofilm formation, and propensity for resistance development. Furthermore, we elucidate its multifaceted mechanism of action, encompassing both bacterial membrane disruption and induction of oxidative stress, alongside its capacity to enhance host immunity in *Carassius auratus gibelio*. This work provides a scientific foundation for developing ALE as a sustainable and effective alternative to conventional antibiotics in aquaculture.

## 2. Results

### 2.1. In Vitro Antibacterial Activity of ALE Against A. hydrophila

#### 2.1.1. Antibacterial Effect of ALE

**Figure 2:**
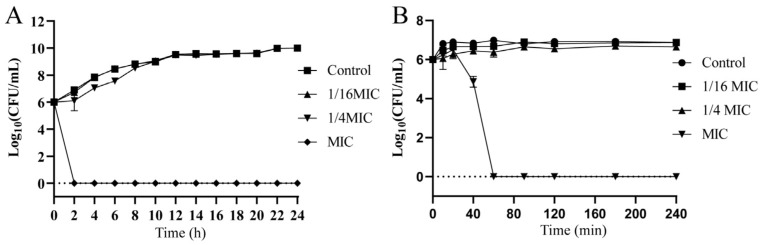
(**A**) Growth curve of ALE against *A. hydrophila*. (**B**) Time-kill curve of ALE against *A. hydrophila*. The data were presented as the mean ± SD (*n* = 3) of three independent experiments.

Broth dilution assays showed that the MIC and MBC values of ALE against *A. hydrophila* were both 8 μg/mL, indicating its potent antibacterial efficacy. As shown in Figure 2A, treatment with ALE at the MIC completely suppressed bacterial growth throughout the observation period. Furthermore, time-kill kinetics analysis revealed that ALE exhibited rapid bactericidal activity, achieving complete bacterial eradication within 1 h (Figure 2B).

#### 2.1.2. The Resistance of *A. hydrophila* to ALE

**Figure 3:**
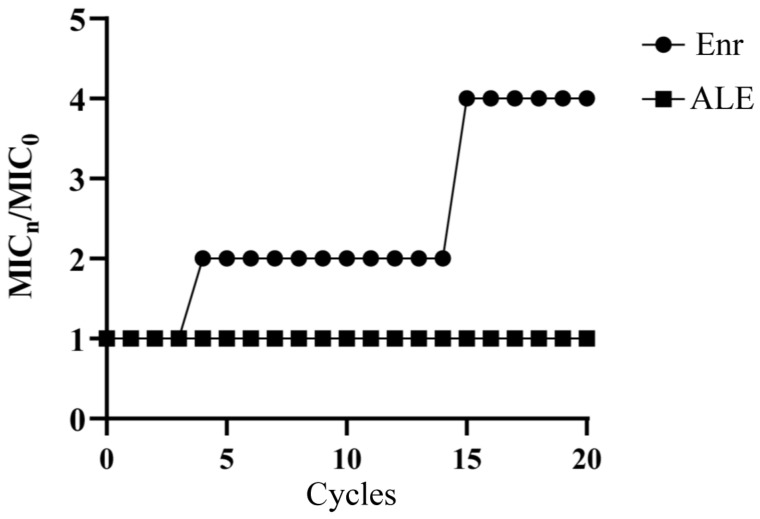
Bacterial resistance study of ALE against *A. hydrophila.* MIC_0_ represents the MIC of ALE against the primary *A. hydrophila*, and MIC_n_ represents the MIC value of ALE against *A. hydrophila* at the nth passage.

The potential for *A. hydrophila* to develop resistance to ALE was assessed through a 20-passage serial exposure experiment and compared with the control antibiotic enrofloxacin. The MIC of ALE remained constant at 8 μg/mL throughout the entire passage series, indicating no detectable development of resistance. In contrast, the MIC of enrofloxacin against the serially passaged bacteria showed a progressive and significant 4-fold increase (from 2 μg/mL 8 to μg/mL) by the 20th passage. These results demonstrated that whereas *A. hydrophila* rapidly develops enrofloxacin resistance, its propensity for resistance development against ALE is significantly lower (Figure 3).

### 2.2. ALE Inhibits Biofilm Formation and Disrupts Biofilms

**Figure 4:**
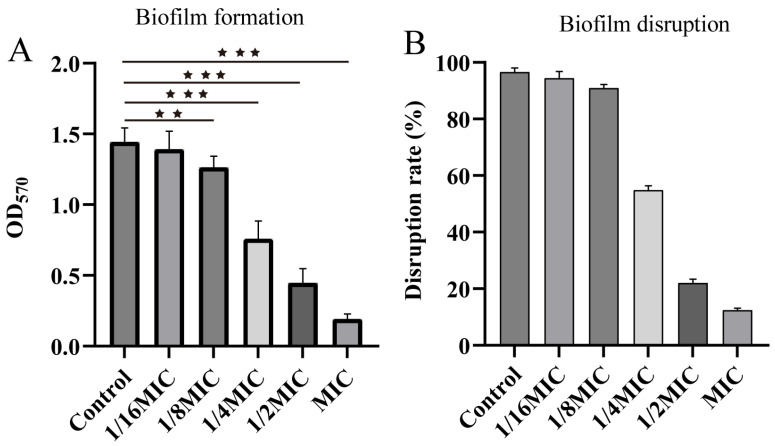
(**A**) Inhibitory effect of ALE on the biofilm formation of *A. hydrophila*. (**B**) Disrupting effect of ALE on the formed biofilms of *A. hydrophila*. The data were presented as the mean ± SD (*n* = 3) of three independent experiments. ^★★^ *p* < 0.01, ^★★★^ *p* < 0.001.

ALE demonstrated a significant and concentration-dependent inhibitory effect on biofilm formation in *A. hydrophila*. The results demonstrated that 1/4 MIC and 1/2 MIC concentrations reduced biofilm formation by approximately 35% and 85%, respectively (Figure 4A), compared to the untreated control. Furthermore, ALE also exhibited significant disruptive effects on pre-formed biofilms. This disruptive effect was significantly enhanced with increasing drug concentrations. A biofilm destruction rate of 90% was achieved at the MIC (Figure 4B). These results indicate that ALE not only prevents biofilm formation but also effectively disrupts mature biofilm.

### 2.3. ALE Induces Oxidative Stress and Membrane Damage in A. hydrophila

#### 2.3.1. ALE Induces Oxidative Stress

**Figure 5:**
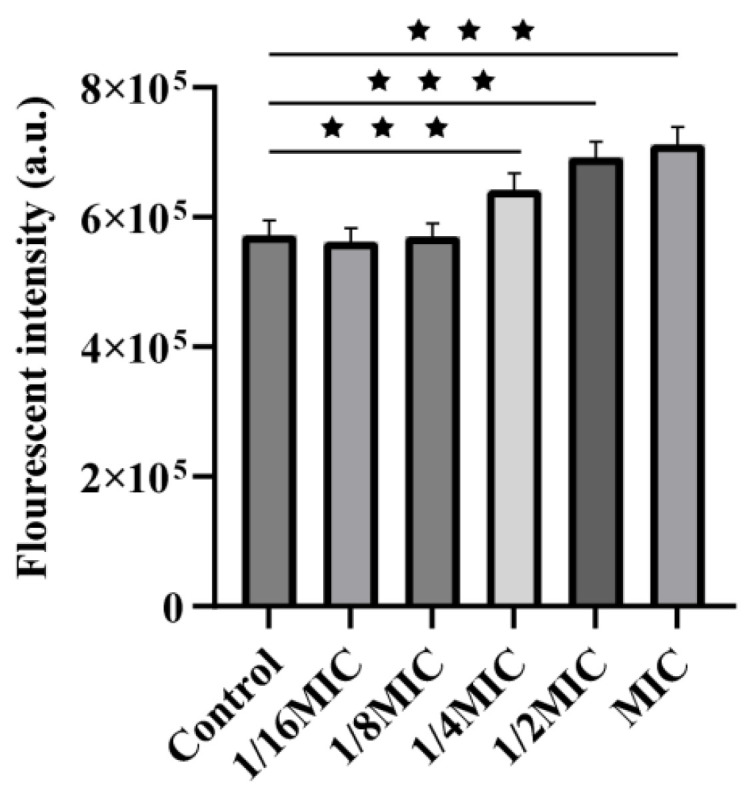
Effect of ALE treatment on ROS accumulation in *A. hydrophila*. The data were presented as the mean ± SD (*n* = 3) of three independent experiments. ^★★★^ *p* < 0.001.

The intracellular ROS levels in *A. hydrophila* were significantly elevated upon ALE treatment in a clear dose-dependent manner. Compared to the untreated group, the relative fluorescence intensity increased by 1.1-fold, 1.2-fold, and 1.3-fold in cells treated at concentrations of 1/4 MIC, 1/2 MIC, and MIC, respectively (Figure 5). These results demonstrated that ALE caused a significant accumulation of ROS in the *A. hydrophila*, leading to oxidative stress, which is likely a key mechanism contributing to its antibacterial activity.

#### 2.3.2. ALE Disrupts the Membrane Structure of *A. hydrophila*

**Figure 6:**
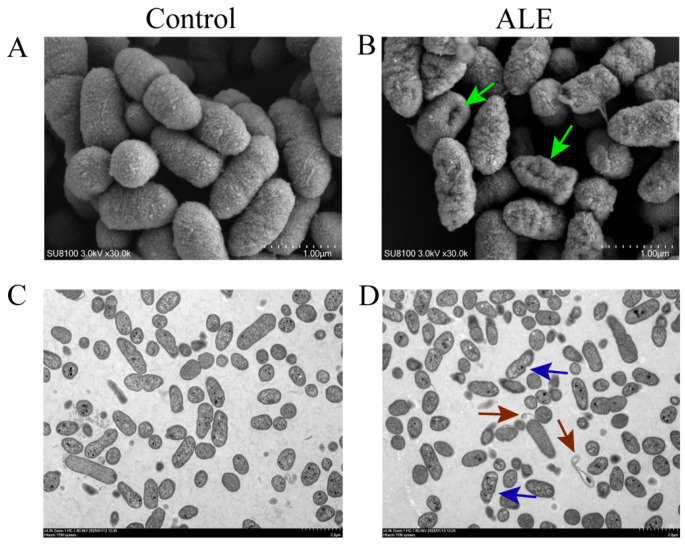
Morphology of *A. hydrophila*. SEM image of control (**A**) and ALE treatment (**B**). TEM image of control (**C**) and ALE treatment (**D**). Green arrows indicate damaged cell structures; red arrows indicate cellular vacuoles; blue arrows indicate damaged cell surfaces.

The membrane damage induced by ALE was observed using electron microscopy. As shown in (Figure 6A,B), scanning electron microscopy (SEM) images revealed that control group bacteria maintained intact cell membrane structures with regular morphology and smooth surfaces. In contrast, ALE-treated cells displayed pronounced membrane structural damage, characterized by conspicuous and surface irregularities (green arrow). Transmission electron microscopy (TEM) further confirmed the damage of membrane structure. Control cells showed densely structured cytoplasmic matrices (Figure 6C). However, ALE-treated cells exhibited a distinct intracellular void (Figure 6D, red arrow) and membrane damage (Figure 6D, blue arrows). These changes in morphological characteristics demonstrated that ALE disrupts the structural integrity of the cell membrane of *A. hydrophila*.

**Figure 7:**
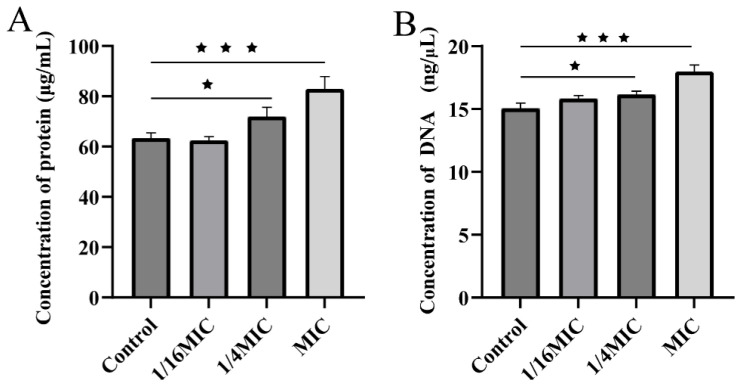
(**A**) Protein leakage from *A. hydrophila* following treatment with ALE. (**B**) DNA leakage from *A. hydrophila* following treatment with ALE. The data were presented as the mean ± SD (*n* = 3) of three independent experiments. ^★^ *p* < 0.05, ^★★★^ *p* < 0.001.

The integrity of the bacterial cell membrane was further assessed by measuring the release of intracellular components. Treatment with ALE at the MIC (8 μg/mL) resulted in a significant leakage of macromolecules. Specifically, the extracellular concentrations of protein and DNA increased to 1.4-fold and 1.2-fold, respectively, compared to the untreated control (Figure 7A,B). This leakage of cellular contents provides direct biochemical evidence that ALE compromises membrane integrity, leading to cell death.

### 2.4. Protective Efficacy of ALE in Carassius auratus gibelio

#### 2.4.1. ALE-Enriched Diet Improves the Survival of *A. hydrophila*-Infected *Carassius auratus gibelio*

To evaluate the protective effect of dietary ALE against *A. hydrophila* infection, a preventive feeding trial was conducted. Gibel carp were fed either a basal diet (control and model groups) or diets supplemented with ALE at 16 mg/kg (low dose) or 32 mg/kg (high dose) for 7 days. After the feeding period, fish in the model and treatment groups were intraperitoneally injected with 100 μL of *A. hydrophila* suspension (1 × 10^7^ CFU/mL), while the control group received an equal volume of sterile PBS. Mortality was monitored for three days post-challenge.

**Figure 8:**
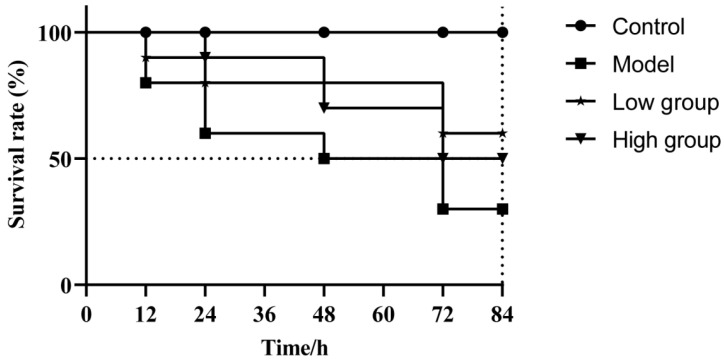
Survival curve following *A. hydrophila* infection. The data are presented as the mean ± SD (*n* = 10). Control = normal diet, healthy untreated; model = normal diet, infected untreated; low group = diet containing 16 mg/kg ALE, infected untreated; high group = diet containing 32 mg/kg ALE, infected untreated.

As shown in Figure 8, mortality was observed in both the model group and the ALE-treated groups at 12 h. At the end of observation period, the survival rate was only 30% in the model group. Dietary supplementation with ALE significantly enhanced host resistance, resulting in a survival rate of 60% and 50% in the low group (16 mg/kg) and high group (32 mg/kg), respectively. It is noteworthy that the low group exhibited a higher survival rate than the high group.

#### 2.4.2. ALE Effectively Reduces Bacterial Loads In Vivo

**Figure 9:**
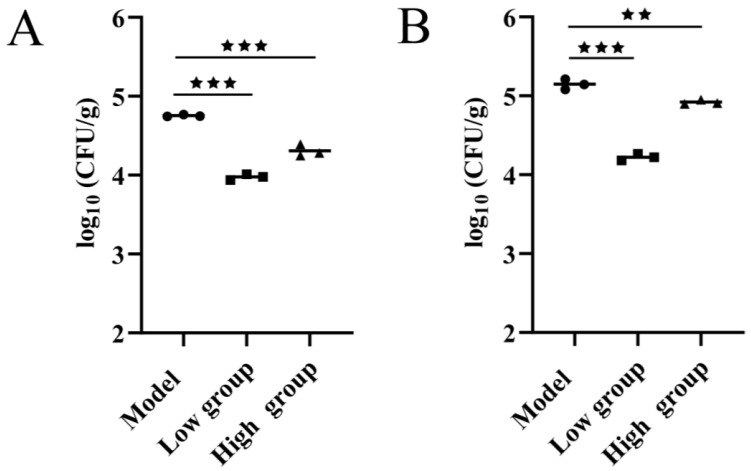
(**A**) Bacterial load in the liver. (**B**) Bacterial load in the kidney. The data are presented as the mean ± SD (*n* = 3) ^★★^ *p* < 0.01, ^★★★^ *p* < 0.001. Model = normal diet, infected untreated; low group = diet containing 16 mg/kg ALE, infected untreated; high group = diet containing 32 mg/kg ALE, infected untreated.

The in vivo antibacterial efficacy of ALE was directly assessed by quantifying bacterial loads in major tissues post-infection. Compared to the model group, the bacterial loads of dietary supplementation with ALE were significantly reduced in both the liver and kidney. The low group exhibited a 6.31-fold and 7.94-fold reduction in the liver and kidney, respectively (Figure 9A). The high group showed a 3.16-fold and 1.58-fold reduction in the liver and kidney, respectively (Figure 9B). These results demonstrated that ALE inhibits the proliferation of *A. hydrophila* in host tissues, particularly at the lower dose.

#### 2.4.3. ALE Attenuates Bacteria-Induced Inflammatory Responses

**Figure 10:**
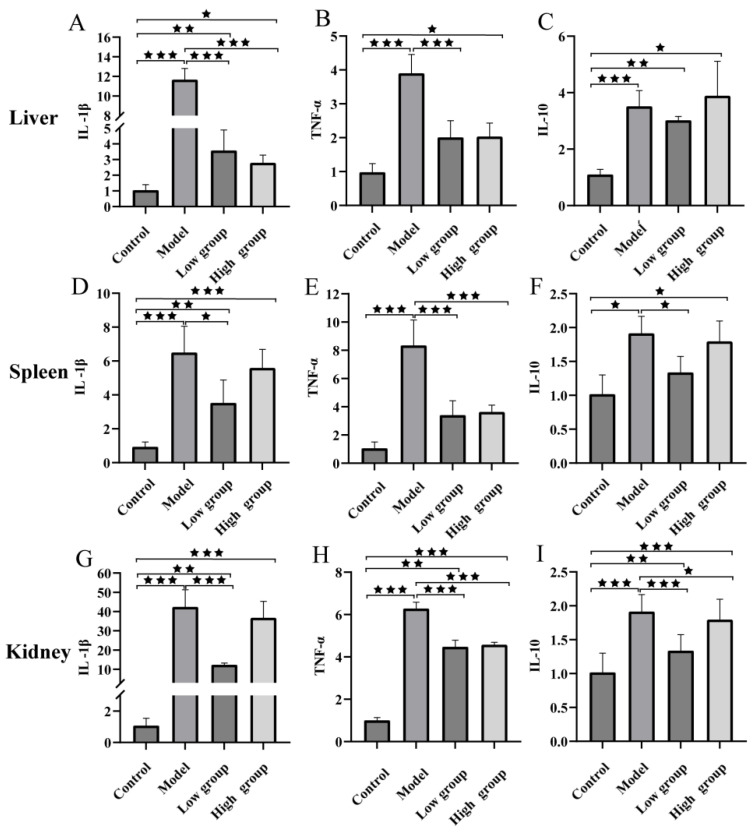
The expression level of the IL-1β (**A**,**D**,**G**), TNF-α (**B**,**E**,**H**), and IL-10 (**C**,**F**,**I**) genes in the liver, spleen, and kidney tissues. The data are presented as the mean ± SD (*n* = 6) of three independent experiments. ^★^ *p* < 0.05, ^★★^ *p* < 0.01, ^★★★^ *p* < 0.001. Groups not compared show no difference. Control = normal diet, healthy untreated; model = normal diet, infected untreated; low group = diet containing 16 mg/kg ALE, infected untreated; high group = diet containing 32 mg/kg ALE, infected untreated.

To evaluate the immunomodulatory effect of ALE, the expression levels of key immune-related genes were analyzed in fish tissues. *A. hydrophila* challenge significantly upregulated the expression of pro-inflammatory cytokines (IL-1β and TNF-α) and an anti-inflammatory cytokine (IL-10) in the liver, spleen, and kidney of fish at the 6 h mark compared to the control group. However, treatment with ALE resulted in a downregulation of the expression of these genes across all examined tissues compared to the model group (Figure 10). These results indicate that ALE helps alleviate the infection-induced inflammatory response.

#### 2.4.4. ALE Improves Immune Responses in Infected Fish

**Figure 11:**
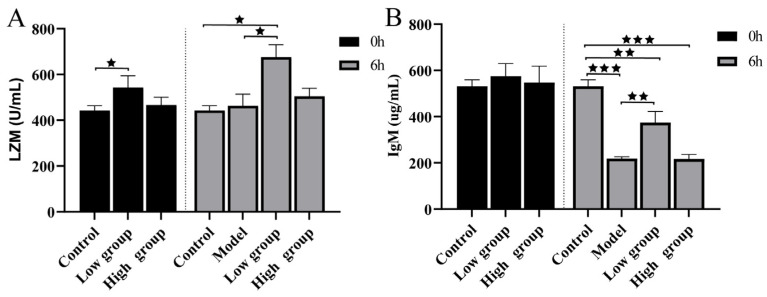
(**A**) The concentration of LZM in serum. (**B**) The concentration of lgM in serum. All experiments were performed in triplicate. The data are presented as the mean ± SD (*n* = 6). ^★^ *p* < 0.05, ^★★^ *p* < 0.01, ^★★★^ *p* < 0.001. Groups not compared show no difference. 0 h: control = normal diet; low group = diet containing 16 mg/kg ALE; high group = diet containing 32 mg/kg ALE. 6 h: control = normal diet, healthy untreated; model = normal diet; infected untreated; low group = diet containing 16 mg/kg ALE, infected untreated; high group = diet containing 32 mg/kg ALE, infected untreated.

The effect of ALE on immune factors was assessed. At 6 h post-challenge, fish fed the low-dose ALE diet (16 mg/kg) exhibited significantly higher activity of LZM, a key bactericidal enzyme of innate immunity, compared to the model group (Figure 11A). Meanwhile, although the infection significantly suppressed IgM levels overall, the low-dose ALE group maintained significantly higher IgM concentrations than the model group (Figure 11B), indicating a supportive effect on the specific antibody response.

#### 2.4.5. ALE Improves Infection-Induced Oxidative Stress

**Figure 12:**
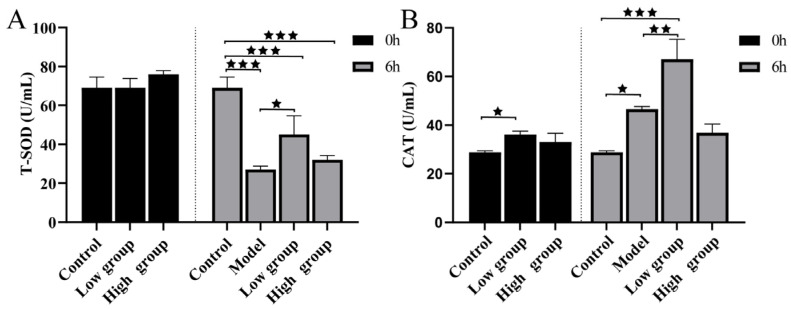
The activity of T-SOD (**A**) and CAT (**B**) in serum. The data are presented as the mean ± SD (*n* = 6). ^★^ *p* < 0.05, ^★★^ *p* < 0.01, ^★★★^ *p* < 0.001. Groups not compared show no difference. 0 h: control = normal diet; low group = diet containing 16 mg/kg ALE; high group = diet containing 32 mg/kg ALE. 6 h: control = normal diet, healthy untreated; model = normal diet, infected untreated; low group = diet containing 16 mg/kg ALE, infected untreated; high group = diet containing 32 mg/kg ALE, infected untreated.

The antioxidant capacity of fish was evaluated by measuring key enzyme activities. The bacterial challenge led to a significant reduction in T-SOD activity in the model group. However, ALE treatment, particularly at the low dose (16 mg/kg), significantly alleviated this suppression (Figure 12A). Similarly, the activity of CAT was significantly higher in the low-dose ALE group compared to the model group (Figure 12B). These results demonstrate that ALE enhances the antioxidant defense system in infected fish.

#### 2.4.6. ALE Alleviates Histopathological Damage

**Figure 13:**
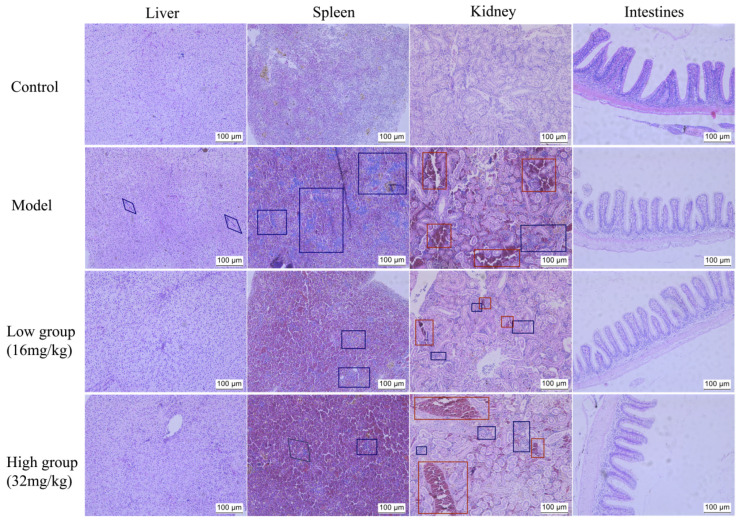
H&E-stained sections of major tissues from *C. auratus gibelio* challenged with *A. hydrophila.* Scale bar: 100 μm. The blue boxes indicate inflammatory cell infiltration, and the red boxes indicate cellular hemolysis. Control = normal diet, healthy untreated; model = normal diet, infected untreated; low group = diet containing 16 mg/kg ALE, infected untreated; high group = diet containing 32 mg/kg ALE, infected untreated.

Following bacterial challenge, the liver, spleen, kidney, and intestinal tissues from the control group exhibited normal histological architecture. In contrast, the model group pronounced pathological changes, including inflammatory cell infiltration in the liver, spleen and kidney (Figure 13, blue square), as well as significant hemorrhage in the kidney (Figure 13, red square). Treatment with ALE markedly reduced the area of inflammatory cell infiltration in the liver, spleen, and kidney tissues. Furthermore, kidney tissue hemorrhage was markedly alleviated in the low group (16 mg/kg), whereas no significant improvement was observed in the high group (32 mg/kg).

#### 2.4.7. Safety Assessment of ALE-Enriched Diets

**Figure 14:**
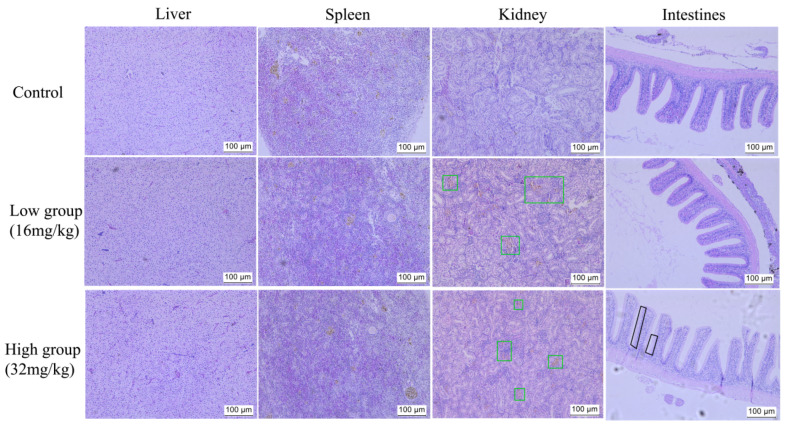
H&E-stained sections of major tissues in *C. auratus gibelio* after ALE treatment. Scale bar: 100 μm. Control = normal diet; low group = diet containing 16 mg/kg ALE; high group = diet containing 32 mg/kg ALE).

Histopathological examination was conducted to evaluate the safety of ALE-enriched diets after one week of administration. Tissues from the control group showed normal histological architecture. In ALE-treated groups, the liver and spleen exhibited no significant morphological alterations compared to the control. However, hemosiderin deposition in the kidney was observed in both treatment groups (Figure 14, green square). In intestinal tissue, no remarkable changes were detected in the low group (16 mg/kg), whereas a noticeable increase in the number of goblet cells was evident in the high group (32 mg/kg) (Figure 14, black square).

## 3. Discussion

Bacterial pathogens pose a severe and persistent threat to the aquaculture industry, among which *A. hydrophila* is one of the most prevalent and devastating [21,22,23,24]. The prolonged and indiscriminate use of antibiotics has led to the emergence of severe resistance in *A. hydrophila*, highlighting the urgent need for novel therapeutic agents. In this context, plant extracts have gained increasing attention as green, efficient, and low-toxicity alternatives in aquaculture, recognized for their capacity to promote growth, strengthen immune responses, and enhance disease resistance in fish [25,26]. Among them, ALE, an allicin derivative, has demonstrated potent antibacterial activity. Although previous research had indicated that ALE can inhibit spore germination and hyphal development of *Penicillium chrysogenum*, its antibacterial activity against *A. hydrophila* and the underlying mechanisms remained unclear [20]. Therefore, the present study systematically investigated the antibacterial effects of ALE against *A. hydrophila* through a combination of in vivo and in vitro experiments.

This study first evaluated the in vitro antibacterial effect of ALE against *A. hydrophila*. At the MIC concentration, ALE not only effectively inhibited bacterial growth but also exhibited rapid bactericidal activity, achieving 99.9% reduction in viable cells within 1 h. This rapid and highly efficient antibacterial property significantly reduces the time for bacterial adaptive responses, thus substantially lowering the risk of bacterial resistance. Consistently, subsequent resistance induction assays confirmed that the MIC of ALE remained unchanged after 20 consecutive passages under sub-inhibitory concentration (1/2 MIC) pressure, while the control of enrofloxacin showed a 4-fold increase under the same conditions. These results indicated that ALE possesses potent and rapid bactericidal activity with a low potential for inducing resistance in *A. hydrophila*.

Biofilm formation represents a key adaptive strategy through which bacteria, including *A. hydrophila*, enhance their pathogenicity and environmental persistence. These multicellular aggregates are notoriously difficult to eradicate and contribute significantly to treatment failure and chronic infections [27,28]. In this study, ALE demonstrated a remarkable dual anti-biofilm ability. The results demonstrated that ALE possesses both biofilm inhibition and eradication capabilities. At the MIC, biofilm formation was suppressed by more than 90%. Notably, even at a sub-inhibitory concentration (1/4 MIC), ALE eradicated over 50% of pre-formed biofilms. This dual potent effect to both inhibit biofilm formation and eradicate pre-formed biofilms underscores the therapeutic potential of ALE in controlling persistent infections caused by *A. hydrophila*.

Based on the favorable antibacterial activity of ALE against *A. hydrophila*, the antimicrobial mechanism of ALE was further investigated. Our data indicated that the antibacterial process is likely initiated by a rapid, dose-dependent accumulation of intracellular ROS, a hallmark of oxidative stress. The surge in ROS levels suggests that the induction of oxidative stress is a primary event [29,30]. This ROS burst subsequently inflicts severe damage to the cell membrane, as directly evidenced by SEM and TEM showing membrane disruption and leakage of intracellular contents. Therefore, this finding supported a mechanistic model wherein ALE triggers substantial ROS accumulation, which plays a pivotal role in mediating irreversible membrane damage and ultimately cell death through the loss of cytoplasmic components.

The potent in vitro antibacterial efficacy of ALE prompted us to evaluate its efficacy in a live host system using *C. gibelio*. Consistent with previous reports on the benefits of phytogenic supplements in aquaculture, dietary incorporation of ALE conferred significant protection against *A. hydrophila* challenge. In this study, an acute infection model was successfully established in fish by intraperitoneal injection of *A. hydrophila* suspension (1 × 10^7^ CFU/mL), which induced significant infection symptoms and pathological responses within a short period. The low dose of ALE (16 mg/kg) significantly increased the survival rate of infected fish. Furthermore, ALE treatment led to a marked reduction in bacterial loads in key tissues such as the liver and kidney, providing direct evidence of its in vivo antibacterial action. These results demonstrate that ALE provides effective protection of *C. gibelio* against *A. hydrophila* infection.

The host immune response to bacterial invasion involves a complex interplay of inflammatory mediators [31]. In this study, infection with *A. hydrophila* triggered a strong inflammatory response in *C. gibelio*, characterized by significant upregulation of pro-inflammatory cytokines (IL-1β, TNF-α) and the anti-inflammatory cytokine IL-10 across multiple tissues. The low group (16 mg/kg) showed a marked downregulation in the expression of these genes (*p* < 0.05 vs. model) compared to the model group, suggesting that ALE alleviates infection-induced hyperinflammation and helps restore immune homeostasis. High-dose ALE (32 mg/kg) showed a different pattern: it slightly downregulated IL-1β in the liver but upregulated it in the spleen and kidney back toward model levels. TNF-α showed no significant changes between doses. Notably, IL-10 was significantly upregulated by the high dose in all tissues, returning to model group levels. This suggests that high-dose ALE may trigger a compensatory anti-inflammatory response while failing to maintain the broad suppression achieved by the low dose, consistent with the non-linear dose–response observed in survival and bacterial load data.

Concurrently, ALE enhanced key components of humoral immunity and antioxidant defense. Lysozyme (LZM) is a key bactericidal enzyme of the innate immune system that cleaves bacterial peptidoglycan and promotes phagocytosis [32]. LZM activity is widely used as a reliable marker of non-specific immune status in fish, as it reflects the activation state of phagocytes and is readily modulated by immunostimulatory feed additives [33]. In our study, LZM activity was significantly enhanced in the low group, reinforcing innate bacterial clearance capacity. Notably, at 0 h, no significant differences in LZM activity were observed between any groups (control, model, low, or high), confirming that the basal immune status was comparable across all treatment groups before bacterial challenge (Figure 12A).

Regarding IgM, this immunoglobulin is the predominant antibody in fish systemic immunity and plays a critical role in opsonization and complement activation against bacterial pathogens [34]. At 0 h, serum IgM concentrations were similar across all groups, indicating no pre-existing differences in humoral immune status. Following *A. hydrophila* infection, the intense infection suppressed IgM levels (model group). In contrast, the low ALE-treated group maintained significantly higher IgM concentrations, suggesting a supportive effect on the specific antibody response amidst inflammatory stress.

Infection also imposed significant oxidative stress, as evidenced by perturbations in the antioxidant defense system. The high bacterial challenge led to a substantial depletion of T-SOD activity, likely overwhelmed by excessive ROS generated during the respiratory burst [35]. In response, CAT activity was elevated, representing a compensatory mechanism to eliminate accumulating H_2_O_2_ [36]. Critically, ALE treatment effectively ameliorated this oxidative burden. Fish receiving the low-dose ALE diet maintained significantly higher T-SOD activity and exhibited a more robust CAT response compared to the infected model group, demonstrating that ALE enhances the overall antioxidant capacity to mitigate infection-induced oxidative damage. These results collectively demonstrate that ALE not only modulates inflammatory signaling but also supports humoral immunity and enhances antioxidant capacity, contributing to a more effective host defense against *A. hydrophila* infection.

Histopathological analysis provided direct evidence for the protective efficacy and safety of ALE. Following bacterial challenge, fish receiving the low-dose ALE (16 mg/kg) demonstrated significantly attenuated inflammatory responses across multiple tissues and a notable reduction in renal hemorrhage compared to the infected model group, indicating that ALE can alleviate bacteria-induced inflammatory responses and exert favorable tissue-protective effects. In summary, ALE can reduce the bacterial load in fish to alleviate the inflammatory response caused by *A. hydrophila* and mitigate fish tissue damage. Meanwhile, the increased LZM content before challenge (0 h) indicates that ALE may enhance the immune regulation of fish.

Importantly, one-week dietary administration of ALE revealed that the low dose (16 mg/kg) did not induce significant pathological alterations. However, the high group exhibited increased hemosiderin deposition in renal tissue, suggesting potential erythrocyte alterations, alongside an increase in intestinal goblet cell numbers, which may represent a mucosal adaptive or irritant response. These results confirm that the low dose (16 mg/kg) exhibits high safety. Meanwhile, the high dose (32 mg/kg) showed certain cytotoxicity, which may explain why the therapeutic effect in the low dose (16 mg/kg) was better than that in the high dose (32 mg/kg).

## 4. Materials and Methods

### 4.1. Bacterial Strain and Chemicals

*A. hydrophila* 823 was isolated from diseased blunt snout bream Megalobrama amblycephala at a fish farm in Yixing, China. The bacterial strain was streaked onto LB agar plate (Luria–Bertani medium, Qingdao, China) and incubated overnight until single colonies appeared. Then, a single colony was inoculated into LB liquid broth and cultured at 28 °C with shaking at 180 rpm for 20 h to prepare the bacterial suspension for subsequent experiments.

ALE (>98% purity) was obtained from Landcent (Shanghai, China). Enrofloxacin was obtained from Aladdin (Shanghai, China). Dimethyl sulfoxide (DMSO), agar, NaCl, methanol, glutaraldehyde, paraformaldehyde, ethanol, and glacial acetic acid were purchased from Sinopharm Chemical Reagent (Beijing, China). Tricaine methanesulfonate (MS-222), crystalline violet, xylene, hematoxylin, and eosin were purchased from Solarbio life science (Beijing, China). 4′,6-diamidino-2-phenylindole (DAPI) and propidium iodide (PI) were bought from Yeasen BioTechnologies Co., Ltd. (Shanghai, China). SYTOX Green was bought from Invitrogen (Carlsbad, CA, USA).

### 4.2. In Vitro Antibacterial Assay

#### 4.2.1. Determination of Minimum Inhibitory Concentration (MIC) and Minimum Bactericidal Concentration (MBC)

The bacterial strain was inoculated into LB liquid medium and cultured in a shaker incubator (Suzhou Jimei Electronic Co., Ltd., ZQTY-70 S, Suzhou, China) for 16 h (28 °C, 180 rpm), then diluted to a final concentration of 1 × 10^6^ CFU/mL. A stock solution of ALE at 128 μg/mL was prepared in 1% (*v*/*v*) DMSO and serially 2-fold diluted. Next, 100 μL of each dilution and 100 μL of bacterial suspension were added to a 96-well plate. The wells containing only LB broth were used as negative control, and bacteria with 1% DMSO were used as a positive control. Finally, the plate was incubated at 28 °C for 20 h, and the results were assessed visually. The MIC was defined as the lowest concentration of ALE that completely inhibited visible bacteria growth. The determination of MIC was repeated three times.

Following the determination of the MIC, 50 μL aliquots from wells were spread onto an LB agar plate. After incubation at 28 °C for 20 h, the minimum concentration that yielded no bacterial colonies was defined as the MBC.

#### 4.2.2. Effect of ALE on the Growth and Time-Kill Kinetics of *A. hydrophila*

The growth curve of *A. hydrophila* in the presence of ALE was determined by the plate colony counting method. Briefly, ALE was introduced into 5 mL of LB liquid medium to achieve final concentrations corresponding to the MIC (8 μg/mL), 1/4 MIC (2 μg/mL), and 1/16 MIC (0.5 μg/mL), respectively. The bacteria with 1% DMSO as were used as a positive control. The bacterial suspension was adjusted to 5 × 10^7^ CFU/mL. Then, 100 μL of the bacterial suspension was aseptically transferred into the drug-containing broth and cultured at 28 °C with shaking at 180 rpm. At 2 h intervals, 50 μL aliquots from appropriate dilutions were spread onto LB agar plates and incubated at 28 °C for 20 h. Colonies were counted manually after incubation. The experiment was performed in triplicate.

The time-kill curve of *A. hydrophila* treated with ALE was determined by the plate colony counting method. Briefly, ALE was added to 5 mL of LB liquid medium to achieve final concentrations to the MIC (8 μg/mL), 1/4 MIC (2 μg/mL), and 1/16 MIC (0.5 μg/mL). The bacteria suspension with 1% DMSO served as the positive control. The bacterial suspension was adjusted to 5 × 10^7^ CFU/mL. Then, 100 μL of this suspension was aseptically transferred into the drug-containing broth and cultured at 28 °C with shaking at 180 rpm. Samples were collected at time points of 10, 20, 40, 60, 90, 120, 180 and 240 min. Following serial dilution, 50 μL aliquots from appropriate dilutions were spread onto LB agar plates and incubated at 28 °C for 20 h. Colonies were counted manually after incubation. The experiment was performed in triplicate.

#### 4.2.3. Induction of Bacterial Resistance

The potential for *A. hydrophila* to develop resistance to ALE was investigated and compared with enrofloxacin using a serial passaging method. Initially, the MICs of both ALE and enrofloxacin against *A. hydrophila* were determined. A single bacterial colony was inoculated into liquid medium containing 1/2 MIC concentrations of the respective agent and cultured at 28 °C with shaking at 180 rpm for 20 h. Subsequently, bacteria were inoculated onto fresh LB plates containing each drug with sub-inhibitory concentration (1/2 MIC) and incubated at 28 °C for 20 h. This sequential passage in liquid and solid media was defined as one cycle. The process was repeated for 20 cycles. Upon observation of new colony growth after each complete cycle, the MICs of the passaged bacteria were re-determined by the microdilution method.

### 4.3. Antibacterial Mechanism of ALE Against A. hydrophila

#### 4.3.1. Effects of ALE on Biofilm Inhibition and Eradication

The inhibitory effect of ALE on biofilm formation by *A. hydrophila* was measured using the crystal violet staining method. Briefly, the concentration of bacterial suspension was adjusted to 1 × 10^6^ CFU/mL. A stock solution of ALE (32 μg/mL) was prepared in 1% DMSO and serially diluted by the 2-fold dilution method. Next, 100 μL of each dilution and 100 μL of bacterial suspension were successively added to a 96-well plate. The wells containing only LB broth were served as negative control, and bacteria with 1% DMSO were regarded as positive control. The plate was incubated at 28 °C for 20 h. After incubation, the culture medium was aspirated, and the plate was washed three times with a 0.85% NaCl solution. The biofilms were fixed with 200 μL methanol for 15 min, stained with 1% crystalline violet for 20 min, and gently rinsed under running water. Finally, the stained biofilms were dissolved with 200 μL of a 30% glacial acetic acid solution, and the absorbance was measured at 570 nm using a spectrophotometer (MultiskanGO, Thermo Scientific, Vantaa, Finland). The experiment was performed in triplicate.

The ability of ALE to disrupt pre-formed biofilms was evaluated using the crystal violet staining method. Briefly, a bacterial suspension adjusted to 1 × 10^6^ CFU/mL was added to a 96-well plate and incubated at 28 °C for 20 h. After incubation, the culture medium was carefully aspirated to remove non-adherent cells. Culture medium containing ALE at different concentrations was added, followed by incubation at 28 °C for 20 h; the OD_1%DMSO_/OD_normal growth_ was used as the control group. Then, the treated biofilm was quantified by the crystal violet assay, and the OD_570_ values were tested to calculate the percentage of the residue of the biofilm.

Biofilm disruption rate (%) = [1 − (OD_compound_ − OD_blank_)/(OD_normal growth_ − OD_blank_) × 100].

#### 4.3.2. Determination the Reactive Oxygen Species (ROS) Level in *A. hydrophila*

Intracellular ROS levels in *A. hydrophila* after ALE treatment were measured using a ROS assay kit (Yeasen BioTechnologies Co., Ltd., Shanghai, China). An isolated bacterial colony was transferred to 5 mL LB broth and cultured at 28 °C with shaking until the mid-exponential phase (OD_600_ = 0.3). The bacterial cells were collected by centrifugation (3 min, 6000 rpm), washed twice with the 0.85% NaCl solution and resuspended in a 6 mL 0.85% NaCl solution. The bacterial suspension was divided into six aliquots. Five of them were treated with ALE to achieve final concentrations of 1/16 × MIC, 1/8 × MIC, 1/4 × MIC, 1/2 × MIC and MIC, followed by incubation at 28 °C for 30 min. Bacteria cultured in medium containing 1% DMSO served as the control. Then, the bacteria were collected by centrifugation (3 min, 6000 rpm), washed twice with the 0.85% NaCl solution and resuspended in 1 mL of PBS containing 10 μM DCFH-DA fluorescent probe. The suspensions were incubated in the dark for 15 min, after which the cells were washed again and resuspended in 1 mL of the 0.85% NaCl solution. Finally, the mixture was transferred to a black 96-well plate, and the fluorescence intensity was measured at 488/525 nm excitation/emission wavelengths. The experiment was performed in triplicate.

#### 4.3.3. Effect of ALE Treatment on the Membrane Integrity of *A. hydrophila*

To further verify membrane integrity and the morphological of bacterial cells, SEM and TEM were observed. An isolated colony was transferred to 5 mL LB broth and cultured at 28 °C with shaking until the mid-exponential phase (OD_600_ = 0.3). The solution was then treated with ALE at the MIC for 4 h. Bacteria grown in medium containing 1% DMSO served as the control. The bacterial cells were collected by centrifugation (3 min, 6000 rpm) and washed twice with the 0.85% NaCl solution. The bacterial samples were fixed with 2.5% glutaraldehyde, dehydrated with a graded ethanol series, dried, and coated with gold; then, they were observed under a scanning electron microscope (SEM, SU8100, Hitachi, Santa Clara, CA, USA). The bacterial samples were fixed with 2.5% glutaraldehyde and 1% osmium tetroxide; then, they were dehydrated with a graded ethanol series, embedded in resin, sectioned, stained, and observed under a transmission electron microscope (TEM, HT7800, Hitachi, Santa Clara, CA, USA).

#### 4.3.4. Detection of DNA and Protein Leakage

ALE was added to 50 mL of bacterial culture to final concentrations of 1/16 × MIC, 1/4 × MIC and MIC and cultured at 28 °C with shaking at 180 rpm for 4 h. The cultures were centrifugated to collect the supernatant (10 min, 6000 rpm), which was lyophilized using a freeze dryer (Light Ace HK Ltd., FreeZone 6L, Beijing, China). An amount of 0.2 g lyophilized powder was dissolved in 1 mL of ultrapure water. The DNA concentration was determined by a microspectro-photometer (Thermo, Waltham, MA, USA), while the protein concentration was determined using a BCA Protein Concentration Assay Kit (Nanjing Construction Bioengineering Institute, Nanjing, China). The culture medium alone was used as the background control. The experiment was performed in triplicate.

### 4.4. Protective Effect of ALE on C. auratus gibelio Against A. hydrophila Infection

#### 4.4.1. Experimental Fish and Feeding Regimen

Healthy *C. auratus gibelio* (30 ± 3 g) were obtained from the Nanquan experimental station of the Freshwater Fishery Research Center (Wuxi, China). Experimental feeds were prepared by uniformly blended ALE with the basal diet using soybean oil as a binder to achieve final ALE concentrations of 16 mg/kg and 32 mg/kg. After acclimation for two weeks, fish were randomly assigned to three groups (control, low group, and high group, *n* = 60/group). The fish were fed three times daily at 4% body weight for one week (8:30, 12:30, and 17:00). The water temperature, pH, and dissolved oxygen concentration were maintained at 28–30 °C, 6.5–7.6, and 5.0 mg/L, respectively.

#### 4.4.2. Bacterial Challenge and Sample Collection

Before bacterial challenge, fish were fasted for 24 h, and 10 fish from each group were anesthetized with MS-222 (100 mg/L). Blood was collected from the caudal vein via an EDTA-coated syringe, and liver, kidney, spleen, and intestinal tissues were collected for further analysis.

For the challenge experiment, 20 fish were randomly selected from both the low group and high group and intraperitoneally injected with a 100 μL bacterial suspension (1 × 10^7^ CFU/mL). Fish from the control group were divided into two subgroups: one subgroup (*n* = 20) was injected with a 100 μL 0.85% NaCl solution serving as the negative control (control = healthy untreated), while the other subgroup (*n* = 20) received an equal volume of the bacterial suspension (1 × 10^7^ CFU/mL) as the positive control (model = infected untreated) (Table 1). At 6 h post-challenge, 10 fish were sampled from each treatment group. The fish were anesthetized, and blood was collected from the caudal vein. Meanwhile, liver, kidney, spleen, and intestinal tissues were collected for further analysis. The remaining fish in each group were maintained for a three-day mortality observation.

#### 4.4.3. Activity of Antioxidant Enzyme and Immunity Index

The blood collected from the caudal vein of fish was centrifuged at a low speed (10 min; 2500 rpm), and the supernatant was aspirated. Then, the activities of key antioxidant enzymes, including lysozyme (LZM), total superoxide dismutase (T-SOD) and catalase (CAT), and the immunity index of immunoglobulin M (IgM) were measured using assay kits (Nanjing Construction Bioengineering Institute, China).

#### 4.4.4. RNA Extraction and qRT-PCR Analysis

Total RNA of the liver, spleen, and kidney samples was extracted using the RNAiso Plus reagent (Takara, Beijing, China) following the manufacturer’s instructions. Quantity and quality of the RNA was assessed by OD_260_/OD_280_ using a NanoDrop 2000 (Thermo Scientific, Waltham, MA, USA). The cDNA was synthesized using a HiScript^®^ III RT SuperMiX for qPCR (+gDNA WIper) kit (Vazyme Biotech, Nanjing, China).

Real-time PCR was performed using SYBR qPCR Masret mix (Vazyme, Nanjing, China) on a CFX real-time PCR detection system (Bio-Rad, Hercules, CA, USA). Three technical replicates and a negative control were performed for all samples. The relative expression of target genes (IL-1β, IL-10, and TNF-α) was calculated using the 2^−ΔΔCt^ method, and β-actin was used as the reference gene, The primers (Table 2) were synthesized by Sangon Biotech (Shanghai, China).

#### 4.4.5. Histopathological Analysis by H&E Staining

Before and after challenge, liver, spleen, kidney, and intestinal tissues were fixed in 4% paraformaldehyde solution overnight. Samples were dehydrated with grade ethanol (from 70% to 100%), cleared in xylene, embedded in paraffin, and sectioned at 5 µm. Tissue sections were then stained with hematoxylin–eosin (H&E) according to a standard protocol and then observed under microscope (Leica DM 1000LED, Leica, Düsseldorf, Germany).

### 4.5. Statistical Data Analysis

All experiments were performed in triplicate. The statistical analyses were conducted using SPSS 26, with graphical representations prepared in GraphPad Prism 9.5. Data were expressed as mean ± standard deviation (SD), and differences between groups were analyzed using a *t*-test: *p* < 0.05 represents a significant difference and *p* < 0.01 represents an extremely significant difference.

## 5. Conclusions

This study demonstrated that ALE exhibits potent in vitro antibacterial activity against *A. hydrophila*, with an MIC of 8 μg/mL. ALE exhibited rapid and strong bactericidal activity with a low tendency for resistance development and effective inhibition and eradication of bacterial biofilms. Mechanistic studies revealed that ALE induces intracellular ROS accumulation, which leads to bacterial membrane disruption and subsequent leakage of DNA and proteins, ultimately resulting in cell death. The challenge test of *A. hydrophila* in *C. auratus gibelio* fish demonstrated that ALE has strong protection against *A. hydrophila* infection. A dietary dose of 16 mg/kg significantly reduced bacterial loads in tissues, attenuated inflammatory responses, and alleviated histopathological damage in the fish. These findings support the potential of ALE as a promising phytogenic agent for controlling *A. hydrophila* infections in aquaculture.

## Figures and Tables

**Table 1:** Experimental groups and treatment regimens of animals.

Drug Treatment Group	Different Treatments	Challenge Treatment Group	Different Treatments
Control (*n* = 120)	fed with normal diet	Control (*n* = 20) healthy untreated	100 μL 0.85% NaCl solution
		Model (*n* = 20) infected untreated	100 μL 1 × 10^7^ CFU/mL bacterial suspension
Low group (*n* = 60)	fed with diet containing 16 mg/kg ALE	Low group infected untreated (*n* = 20)	100 μL 1 × 10^7^ CFU/mL bacterial suspension
High group (*n* = 60)	fed with diet containing 32 mg/kg ALE	High group infected untreated (*n* = 20)	100 μL 1 × 10^7^ CFU/mL bacterial suspension

**Table 2:** Sequences of primer pairs used in real-time PCR.

Primer Name	Primer Sequence (5′-3′)	GenBank Accession
IL-1β-F	GCGCTGCTCAACTTCATCTTG	AJ249137 [37]
IL-1β-R	GTGACACATTAAGCGGCTTCAC	
TNF-α-F	CATTCCTACGGATGGCATTTACTT	EU069818 [37]
TNF-α-R	CCTCAGGAATGTCAGTCTTGCAT	
IL-10-F	AGTGAGACTGAAGGAGCTCCG	HQ259106 [38]
IL-10-R	TGGCAGAATGGTGTCCAAGTA	
β-actin-F	CAAGATGATGGTGTGCCAA	AB039726 [37]
β-actin-R	ACCGACCATGACGCCCTGATGT	

## Data Availability

The raw data supporting the conclusions of this article will be made available by the authors on request.
